# How Symbols and Social Interaction Influence the Experienced Utility of Sustainable Lifestyle Guiding Policies: Evidence from Eastern China

**DOI:** 10.3390/ijerph19074305

**Published:** 2022-04-03

**Authors:** Xiu Cheng, Ruyin Long, Fan Wu

**Affiliations:** 1 College of Economics and Management, Nanjing Forestry University, Nanjing 210037, China; rjql_3407@njfu.edu.cn; 2 School of Business, Jiangnan University, Wuxi 214122, China; longruyin@163.com

**Keywords:** symbolic value, social interaction, sustainable lifestyle, policy-experienced utility, regional differences

## Abstract

As the key to mitigating climate change, a sustainable lifestyle has become a focus of environment policy. Past studies have largely neglected the symbols of sustainable lifestyle guiding policies and failed to capture its effect on the experienced utility of sustainable lifestyle guiding policies (*EUSLGP*). To address this drawback, symbolic value was incorporated into a model consisting of social interaction and the *EUSLGP*. With data collected from 3257 respondents in Eastern China, ordinary least squares were applied to examine hypotheses and two-stage least squares based on the instrumental variable to verify the results. Results show that symbolic value combines self-expression value, relationship consolidation value, group identification value, and status-showing value, and is positively associated with *EUSLGP*. Social interaction plays a moderating role in the association between symbolic value and *EUSLGP*. Moreover, significant regional differences are discovered in the identified relationships. Consequently, policy suggestions, covering symbolic value, social interaction, and regional conditions, are proposed to enhance the *EUSLGP* for other countries and regions.

## 1. Introduction

A sustainable lifestyle is the way in which individuals practice habitual or conscious activities to benefit the environment [1]. It is not only a reflection on ways of shopping and daily activities but also related to education, sharing, social events, and identity building [2]. At present, the sustainable lifestyle is changing from a single, strong, state-led action to an orderly, voluntary, public-led action, and policy-experienced utility has become a key factor in realizing this transformation. Policy-experienced utility is the extent to which individuals perceive that a launched policy meets their own needs [3], i.e., money-saving, convenience, and happiness. Accordingly, experienced utility of sustainable lifestyle guiding policies (*EUSLGP*) is the degree to which target groups perceive sustainable lifestyle guiding policies satisfying their actual needs. Policy-experienced utility is the endogenous motivation for individuals to practice the policy, which influences the expected behavior change and policy effect. Studies have revealed that target groups’ responses to sustainable lifestyle guiding policies have been far below expectations. In a more recent survey of Italian, Spanish, and British consumers, all respondents scored lower than the mid-point of a five-point Likert scale concerning food waste [4], suggesting that sustainable lifestyle guiding policies are not fully effective and fail to substantially change residents’ lifestyles. That is to say, the *EUSLGP* is not sufficient to activate target groups to consciously and voluntarily implement policies. Hence, it is highly necessary to identify the contributing factors of *EUSLGP*.

With goods consumption gradually being supplanted by symbol consumption [5], symbolic value has been a key factor when the public evaluates the *EUSLGP* [6]. Symbolic value is the individuals’ subjective value perception regarding self-meaning expression, which is the projection of group members’ self-concept onto the policies. According to role theory, individuals both pursue differences and expect similarities when comparing themselves with others [7]. In terms of policy-experienced utility, the similarities and differences are endowed with some special symbols (i.e., emotion, personality, and status) toward a specific policy. When practicing sustainable lifestyle guiding policies, the actors can convey information of “environmental protection”, “social responsibility”, “health”, and “carbon emission reduction” to their surroundings, and can also obtain emotional resonance with others with the establishment of special interpersonal relationships [8]. For example, the responsibility, vitality, and openness covered by new energy vehicles not only enhance the functional attributes but also increase consumers’ willingness to buy cars [9]. Hence, symbolic value provides a new idea for unlocking policy-experienced utility to encourage individuals to practice sustainable lifestyles.

The influence of social interaction on the *EUSLGP* cannot be neglected when studying public responses to policies. Social interaction refers to a dynamic interaction between different individuals through information dissemination and imitative learning [10]. In essence, it is a process in which humans’ behaviors or feelings are influenced by others. It has been revealed that social interaction influences behavior choices by verbal information, emotion, and norms [11]. Studies have confirmed that the psychological reference generated by social interaction can effectively promote walking or cycling [12], and that the social incentive generated by social comparative information is more effective than the economic incentive [13]. For instance, a survey of 57 million online consumers indicated that over 50% of purchase decisions were affected by other consumer reviews [14]. Furthermore, it was discovered that 70% of consumers regarded the interactive information as important references for decision-making [15]. It can be seen that social interaction has a key influence on the value judgment and behavioral decision-making of individuals. Hence, it is important to study the *EUSLGP* by taking social interaction into account.

The objectives of this study were to discover the effects of symbolic value and social interaction on the *EUSLGP*, and to find targeted ways to promote sustainable lifestyles. ***The major contributions of this study are as follows:*** (1) symbolic value is built as a composite of self-expression value, relationship consolidation value, group identification value, and status-showing value to discover its effect on *EUSLGP*—this fills the gap in implicit values in policy research and provides a new perspective and idea for environmental policy studies; (2) an endogeneity test using an instrumental variable method is proposed to verify the moderating effect of social interaction on the association between symbolic value and *EUSLGP*, thus providing methodological support for policy formulation and policy effect evaluation; (3) regional differences are found in the effects of symbolic value and social interaction on the *EUSLGP*, providing empirical evidence for policymakers to launch differentiated implementation strategies according to local conditions.

The remainder of this paper is organized as follows: Section 2 presents the research hypotheses. Section 3 demonstrates the method and materials used in this study. Results and discussion are presented in Section 4 and Section 5, respectively. Section 6 concludes the study and proposes policy suggestions.

## 2. Theoretical Analysis and Research Hypotheses

### 2.1. The Impact of Symbolic Value on the EUSLGP

#### 2.1.1. The Structure of Symbolic Value

As a projection of self-identity, self-expression, and self-esteem, symbolic value is highly associated with self-concept [16]. In other words, identifying the self-concept is the key to determining the structure of symbolic value. Studies have found that self-concept can be divided into the individual self, relational self, collective self, and social self [17]. The individual self encapsulates individuals’ need to identify their own personality and independence by distinguishing themselves from others. For the relational self, relationship building and maintainence is the focus for an individual. The collective self outlines that individuals exist as a members of certain social groups (e.g., nationality, generation, class) and interact with other such groups. The social self reflects the attainment or desire to attain recognition from others for the reputation and status of one’s social role. Vigneron and Johnson [18] revealed that a brand’s symbolic value includes self-expression and interpersonal maintenance. Muehling et al. [19] identified three kinds of symbolic associations: personal image, group belonging, and social status. Based on previous studies, symbolic value is built as a composite of self-expression value, relationship consolidation value, group identification value, and status-showing value by taking the individual self, relational self, collective self, and social self as the guiding framework.

#### 2.1.2. The Structure of the *EUSLGP*

Studies have discovered that sustainable lifestyle guiding policies are characterized by economic, emotional, and environmental value. In terms of economy, it was found that economic benefits not only enhance individuals’ enthusiasm to participate in environmental protection policies [20,21] but also facilitate the formation of a stable group to implement policies [22,23]. Regarding emotional experience, previous studies have revealed that the independence, passion, and freedom represented by private cars have significantly inhibited the practice of green travel policies [24], and positive emotional intervention can activate an environmental policy response [25]. Environmental value has been widely investigated with respect to environmental protection policies. A prior study has revealed that environmental value is beneficial to the public response toward environment policies [26], providing an intersection between the interest demands of the public and the government. Hence, economic-experienced utility, emotional-experienced utility, and environmental-experienced utility are selected to build the *EUSLGP*. Specifically, economic-experienced utility is value perception in economic aspects, including expenditure, subsidies, and savings. Emotional-experienced utility focuses on positive emotional achievements such as joy, happiness, and pleasant feelings. Environmental-experienced utility is individuals’ subjective judgment on environmental utility—for instance, regarding environmental protection.

#### 2.1.3. Relationship between Symbol Value and the *EUSLGP*

Human beings will creatively establish a relationship with objects through self-identification, making objects become symbols with special meanings [27]. In a symbolic interaction, individuals will form and modify self-concepts through feedback from the outside world or themselves [28], and thus be guided to engage in various social behaviors. The symbolic value of sustainable lifestyle guiding policies can meet the needs of target group members to express themselves and construct interpersonal relations. Hence, the particular symbolic value of sustainable lifestyle guiding policies makes it possible for the target group to establish policy-experienced utility [29]. In addition, a connection between the decision-makers and the policy is built when they tend to accept and occupy the symbolic value from a specific policy. According to the connection theory, the stronger the association between the individuals and the policy, the more likely they are to be satisfied with the symbolic needs met by the policy, and the more conducive this is to an expected behavioral response pattern [30]. Moreover, symbolic values can easily activate the information storage and memory structure of decision-makers for sustainable lifestyle guiding policies [31], so as to achieve self-coordination and self-esteem; thereby, positive attitudes and enhanced decision-making behaviors are achieved.

In previous studies, it has been revealed that more than half of seafood purchasing decisions are motivated by symbolic value [32], and the judgment of target groups on environmental policies is influenced by specific signs, symbols, and labels [33]. This indicates that symbolic value can promote the *EUSLGP*. Specifically, the symbolic value of sustainable lifestyle guiding policies can meet the demands of target groups to express themselves and build relationships, thus enhancing the *EUSLGP*. Consequently, the following hypothesis is proposed:

**Hypothesis** **1** **(H1).** *Symbolic value is positively associated with the EUSLGP.*

### 2.2. The Moderating Effect of Social Interaction

#### 2.2.1. The Structure of Social Interaction

As a way to seek for information, social interaction contributes to psychological comparison, behavioral imitation, and observational learning [34]. In the process of social interaction, individuals’ access to heterogeneous information is affected by structural characteristics, namely the social network topology structure [35]. Studies have found that the most important structural characteristics are the network ties between actors and the network configuration mode, which is usually expressed by the network size and intensity [36]. The former offers decision-makers external conditions (i.e., subject, relationship) to interact with others, while the latter represents the probability of the target group being engaged in social interaction.

In addition, social interaction affects individual behavior mainly through relationship characteristics, among which trust has been proven to be the most important variable [37]. According to trust theory, social interaction generates exchange behavior through trust, and key information such as environmental knowledge, practical skills, and operational knowledge obtained through a strong trust relationship can directly improve policy-experienced utility [38]. Furthermore, the pressure on individuals from social interaction increases as trust improves [39], by which social behaviors such as comparison, learning, and imitation are encouraged indirectly. In accordance with the above, the structural and relationship characteristics of social interaction were examined and social interaction was measured through variables such as network size, interaction intensity, and trust.

#### 2.2.2. Role of Social Interaction

According to bounded rationality, information gained by individuals is incomplete and their ability to calculate is limited. Bounded rationality limits individuals’ behavioral decision-making and provides the possibility for social interaction. That is, the target group members may exchange information with one another to make up for the lack of information due to limited experience and learning ability when evaluating policy-experienced utility. In addition, social interaction leads to social influence and affects individuals’ decisions through informational influence and normative influence [40]. According to reference group theory, individuals always include and contrast themselves with one another psychologically, identify the attitudes, behavior, norms, and values of their groups, and adjust their own behavior to avoid being excluded by other members of the group [41]. Hence, the judgment of target group members on symbolic value and policy-experienced utility is likely to be influenced by other members. The social interaction between individuals will promote convergence and consistency within the target group, resulting in a positive effect on the *EUSLGP*.

Studies have confirmed that policy responses are influenced by information exchange and group demonstration [42], and group interaction is helpful for promoting sustained behavior [43]. Furthermore, it has been confirmed that self-construal is strongly related to social interaction [44]. It can thus be inferred that improving social interaction will improve the policy-experienced utility brought about by symbolic value. Specifically, target group members construct and express themselves through symbolic values. In this process, their needs, such as pursuing individuality, highlighting status, conveying emotion, and expressing belongingness, will be satisfied, and the *EUSLGP* will improve concurrently. Moreover, the underlying mechanism entails social interaction, promoting the connection between symbolic value and policy-experienced utility, and thus activating the latter. On one hand, social interaction contributes to information exchange and resource sharing, enabling decision-makers to obtain more information about symbolic values from outside. On the other hand, a synergy can be created between symbolic values and social interaction. Moreover, the pressure effect of social norms can be generated by social interaction to encourage individuals to engage in sustainable lifestyle guiding policies [45]. Therefore, the following hypothesis is proposed:

**Hypothesis** **2** **(H2).** *Social interaction positively moderates the relationship between symbolic value and EUSLGP.*

## 3. Materials and Methods

### 3.1. Participants and Data Collection

A questionnaire should be determined before the preliminary and formal investigation are carried out. A brief description of the research purpose should be presented on the front page of the questionnaire to address respondents’ concerns. The questionnaire should be structured according to the variables to be measured. Widely used by researchers, the five-point Likert scale has been proven as the most reliable tool to collect data in a questionnaire survey [46]. Consequently, the questionnaire used in this study was designed based on the five-point Likert scale.

From 2 to 28 May 2019, a preliminary investigation was conducted in Nanjing via Wenjuanxing (https://www.wjx.cn/) (accessed on 1 March 2022), an online questionnaire platform widely used by researchers in China. A total of 586 valid questionnaires were collected after deleting 105 invalid ones. A reliability and validity test of the initial scales was carried out using SPSS 22.0, and the results indicated that the questionnaire had good reliability and validity. Furthermore, the feedback of the respondents was fully considered to ensure that each item was easy to understand when the scales were revised.

The formal investigation was conducted through online and paper questionnaires from 8 July 2019 to 10 January 2020. The questionnaire is available in Appendix A. Specially, paper questionnaires were issued in parks, supermarkets, and food markets to collect data from older residents and those who rarely engaged in online surveys. Key variables, such as sustainable lifestyle guiding policies, symbolic value, and social interaction, were explained to respondents before they filled out the questionnaires. Moreover, all respondents were told that the collected data would not be made public. The data obtained from the investigation have been previously applied to study the public response to low-carbon guiding policies. Details of the survey can be found in Cheng et al. [3].

Eastern China was selected as the research area. There were three reasons for this. First, serious environmental pollution has been found in Eastern China, which has captured researchers’ attention [47]. Second, Eastern China was the first region to promote sustainable lifestyles in the country; thus, its residents are more familiar with policies related to sustainable lifestyles [48]. Lastly, with high-level income, residents in these areas are more eager to establish a better living environment [49].

**Figure 1:**
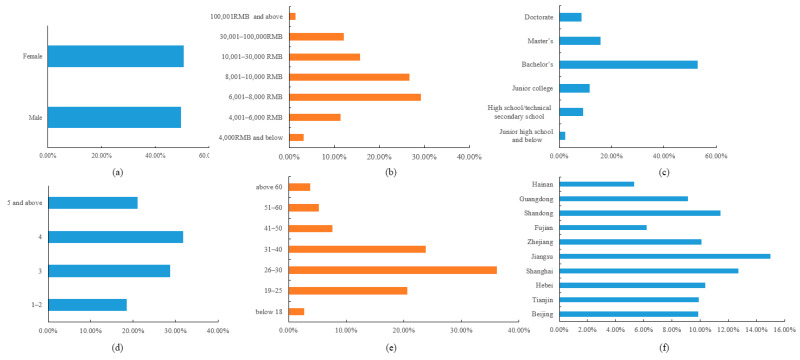
Demographic characteristics of the sample (N = 3257): (**a**) gender, (**b**) monthly income, (**c**) educational level, (**d**) family member, (**e**) age, and (**f**) province.

In total, 3257 valid questionnaires were collected and the respondents’ demographic information is available in Figure 1. The age of the respondents was grouped according to Yang et al. [50]. Of all participants, 50.54% were male and 49.40% were female, and 80.60% of them were aged 19 to 40. Respondents with a bachelor’s degree or above accounted for 77.22%, and 60.49% of the respondents lived in a 3–4-membered family. Moreover, 39.15% of the final sample was from Jiangsu, Shanghai, and Shandong, and 71.78% of the respondents earned between 6000 and 10,000 RMB a month.

### 3.2. Measures and Scale Test

A five-point Likert scale ranging from 1 (“strongly disagree”) to 5 (“strongly agree”) was used to measure symbolic value (*SV*), social interaction (*SI*), and the *EUSLGP*. Symbolic value is measured from self-expression, relationship consolidation, group belonging, and status showing, according to Mandler et al. [51] and Bettels and Wiedmann [52]. A high score means that respondents pay more attention to the symbols extracted form sustainable lifestyle guiding policies. There are sixteen items to evaluate symbolic value; one example is “practicing sustainable lifestyle guiding policies show personality”. Adapted from Chiu et al. [53], questions regarding frequency, ways, and trust relationships were used to measure social interaction. Eleven items were developed to measure this variable. One example is “many people discuss sustainable lifestyle guiding policies with me”. A high score indicates that respondents are more likely to be engaged in sustainable lifestyle guiding policies when interacting with others. According to the structure defined in Section 2.1.2, *EUSLGP* is measured from economic-experienced utility, emotional-experienced utility, and environmental-experienced utility. The items were adapted from Cheng et al. [3] and Herberz et al. [54]. A high score represents a high level of *EUSLGP*. Twelve items are included to measure *EUSLGP*—for example, “practicing sustainable lifestyle guiding policies is an enjoyable behavior”.

An instrumental variable, namely density of gathering with relatives and friends (*DGRF*), was selected for social interaction. The measures of *DGRF* are self-developed with a five-point Likert scale (ranging from 1 “strongly disagree” to 5 “strongly agree”) applied. It consists of three items, and one example is “I visit some relatives and friends regularly”. A high score indicates that respondents are more likely to visit friends and relatives. Furthermore, it was found that demographic variables have effects on pro-environmental behaviors in prior studies. Consequently, demographic variables such as gender, age, educational level, income, and family size were measured at the end of the questionnaire.

The composite reliability (*CR*) and Cronbach’s α were used to test reliability. First, *CR* was greater than 0.79 for all constructs in this study, while no average variance extracted (*AVE*) values were less than 0.5, thus exceeding the respective threshold values for establishing construct reliability [55]. Second, for each construct, the Cronbach’s α values were above the threshold value of 0.7 [56], suggesting ideal internal consistency.

The confirmatory factor analysis was carried out to test the validity of the measurement model using Amos 21.0. The χ^2^/df, good fit index (*GFI*), normed fit index (*NFI*), comparative fit index (*CFI*), incremental fit index (*IFI*), and root mean square error of approximation (*RMSEA*) of the measurement model were 2.452, 0.936, 0.912, 0.954, 0.941, and 0.053, respectively. It can be concluded that all indexes are satisfactory; thereby, the measurement model is valid. The standardized factor loadings of all items were above 0.7 and significantly positive, and no measurement items had double loading, indicating good aggregation validity for each construct [55]. Details of the evaluation index for each construct are included in Appendix B. Furthermore, good discriminant validity can be ensured if the square root of the *AVE* of a construct is greater than the correlation coefficient between the construct and other constructs [57]. As shown in Table 1, this requirement was met for all constructs, confirming that they all have ideal discriminant validity.

### 3.3. Data Analysis Method

#### 3.3.1. Ordinary Least Squares

According to H1 and H2 proposed above, in this study, symbolic value, social interaction, and *EUSLGP* were the independent variable, moderating variable, and dependent variable, respectively. Moreover, demographic variables were set as control variables considering the effects of demographic characteristics on the *EUSLGP*. Ordinary least squares were adopted to test H1 and H2. Based on the model used in Cheng et al. [3], Equation (1) was used to discover the effect of symbolic value on *EUSLGP*.

(1) $$EUSLGP_{i}=\alpha_{10}+\alpha_{11}SV_{i}+\alpha_{12}{\sum Control}_{i}+\varepsilon_{1i}$$

where *i* denotes the *i*-th respondent, “*Control*” refers to control variables, including gender, age, educational level, income, and family size, and *ε*_1*i*_ is the error item.

The interaction item of symbolic value and social interaction was used to test the moderating effect of social interaction on the association between symbolic value and *EUSLGP* [58]. According to Edwards and Lambert [59], Equation (2) was used to quantifiably verify the moderating role of social interaction.

(2) $$EUSLGP_{i}=\alpha_{20}+\alpha_{21}SV_{i}+\alpha_{22}SI_{i}+\alpha_{23}SV_{i}\times SI_{i}+\alpha_{24}\sum Control_{i}+\varepsilon_{2i}$$

where the variables are the same as in Equation (1).

#### 3.3.2. Two-Stage Least Squares

**Figure 2:**
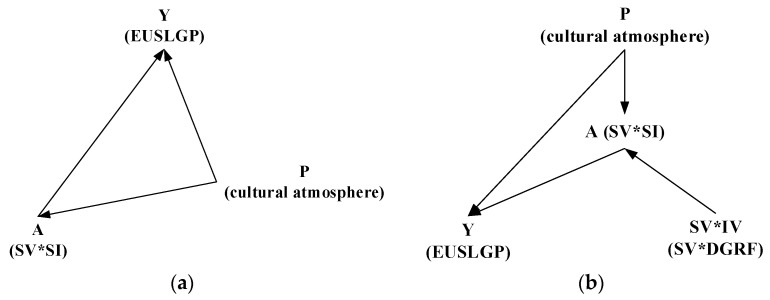
Directed acyclic graphs showing the effects of (**a**) confounder and (**b**) instrumental variable.

In this study, the interaction item of symbolic value and social interaction (SV×SI) was used to verify social interaction’s moderating effect on the association between symbolic value and EUSLGP. It should be noted that unobservable confounders may also influence the results, and thereby endogeneity occurs. It would be problematic if an unobserved confounder P, such as cultural atmosphere, affected both A (SV×SI in this study) and Y (EUSLGP in this study) when the moderating effect was tested. Directed acyclic graphs were used to explain this dilemma according to Kiwanuka-Lubinda et al. [60] and Huntington-Klein [61]. As shown in Figure 2a, two pathways (A→Y and A←P→Y) are available to connect A and Y. According to McElreath [62], the former would be causal while the latter merely builds an association between A and Y. Any changes in A would not influence Y if the second was the only path available. Otherwise, the effect of P would be adjusted alternatively. However, P is unobservable.

An instrumental variable (IV) is useful to estimate the correct causal effect of A on Y. In this study, DGRF was selected as an IV for social interaction, and the interaction item of symbolic value and DGRF (SV×DGRF) was substituted for the interaction item of symbolic value and social interaction (SV×SI). As shown in Figure 2b, SV×DGRF was independent of P, and would not influence Y (EUSLGP) except via SV×SI. In doing so, the causal effect of A that is uncontaminated by unobservable confounders (P) on Y would be identified. A two-stage least squares (2SLS) was built to verify the moderating effect using IV, as demonstrated in Equations (3) and (4):

(3) $$SV_{i}*SI_{i}=\alpha_{30}+\alpha_{31}SV_{i}+\alpha_{32}SI_{i}+\alpha_{33}SV_{i}\times DGRF_{i}+\alpha_{34}\sum Control_{i}+\varepsilon_{3i}\;$$

(4) $$EUSLGP_{i}=\alpha_{40}+\alpha_{41}\hat{SV_{i}\times SI_{i}}+\alpha_{42}SI_{i}+\alpha_{43}SV_{i}+\alpha_{44}\sum Control_{i}+\varepsilon_{4i}\;$$

where DGRF refers to the density of gathering with relatives and friends, and the other variables are the same as in Equation (1).

## 4. Results

### 4.1. Effect of Symbolic Value

With SPSS 22.0 (IBM, New York, USA)used, a normality test was conducted before analyzing the data obtained from questionnaires. Results showed that the absolute values of skewness and kurtosis of all measurement items were less than 2, indicating that the data were approximately normally distributed [63]. Then, Stata 16 Statacorp, Texas, USA) was applied to test the effect of symbolic value on the *EUSLGP* according to Equation (1). As well as the whole sample examined, a group test was conducted by dividing the whole sample into south and north according to the separatrix in China. The following objectives were intended to be achieved by doing so. The first was to test whether there were regional differences in the effect of symbolic value on the *EUSLGP*. The second was the robustness test. The result can be considered valid if the impact effect is detected in different areas. All the results are displayed in Table 2.

The *F*-values of the regression models are 1393.3, 490.2, and 905.6, respectively, suggesting that goodness of fit can be ensured [64]. The regression coefficient of symbolic value and *EUSLGP* is 0.543 for the full sample, significant at the 1% level. This indicates that symbolic value is positively associated with *EUSLGP*, which supports H1. This positive association holds in both the north and south groups, although the regression coefficient is higher in the south (0.575, 30.090) than in the north (0.495, 22.140). Meanwhile, a Chow test conducted to investigate the differences between groups revealed that the positive impact of symbolic value on *EUSLGP* was more significant in the south.

### 4.2. Moderating Effect of Social Interaction

According to Equation (2), the moderating effect of social interaction on the association between symbolic value and *EUSLGP* was examined using the whole sample and the north and south groups. The results are shown in Table 3.

For the full sample, the regression coefficient between *EUSLGP* and symbolic value changed from 0.246 (8.960) to 0.052 (12.650), significant at the 1% level, after adding the interaction term of *EUSLGP* and social interaction. This suggests that social interaction positively moderates the association between *EUSLGP* and symbolic value, thus supporting H2. This moderating effect holds in both the north and south groups, although the coefficient of 12.650 was slightly higher in the south (0.053, 9.860) than in the north (0.051, 8.050). Moreover, a Chow test revealed that the positive moderating effect of social interaction was more significant in the south than in the north.

### 4.3. Endogeneity Test

According to Equations (3) and (4), the *2SLS* method was applied to verify the results. The weak instrumental variable test, Hausman test, and heteroscedasticity test were conducted to make the *2SLS* method more accurate. The *p*-values of these tests’ results meet the requirements of the endogeneity test.

Table 4 presents the results of the endogeneity test. Both *R*^2^ and the *F*-values were large, indicating that the test method was credible and that the instrumental variable had good explanatory power for the endogenous variable. Since the instrumental variable was not weak and all explanatory variables were exogenous, the estimation result of the endogeneity test was consistent with the main estimation result, thus further supporting the moderating effect of social interaction.

## 5. Discussion

### 5.1. The Power of Symbols

#### 5.1.1. Support from Prior Studies

It was found that symbolic value was positively associated with *EUSLGP*, with a regression coefficient of 0.543. This suggests that the symbolic value of implementing sustainable lifestyle guiding policies could significantly improve the experienced utility, consistent with prior research. A study has validated the symbolic attribute of social identity as an effective predictor of purchase intention for electric vehicles [65]. Straus [66] asserted that citizens have the ability to develop and promote the symbolic values contained in policies. These symbolic values reflect citizens’ identification with, requirements for, and expectations of policies, and contain support frameworks for explaining and judging policies, such as knowledge, skills, and social strata. When examining biofuel policies in the United States, Mondou et al. [67] found that the symbolic value embedded in policies was multi-dimensional and tenacious when policies were attacked or not implemented well. In other words, the symbolic value of policies influences the behavior of target groups through symbols such as cultural authority and spiritual power, thus confirming the positive effect of symbolic value on *EUSLGP*.

A study has also revealed that target groups’ attention to the symbolic value of policies has an effect on common policy views and collective policy actions [33], which provided support for the effect of symbolic value on *EUSLGP*. In addition, Pettifor et al. [68] distinguished four attributes in the field of low-carbon innovation: private functional, public functional, private symbolic, and public symbolic. They found that potential mainstream consumers paid more attention to public functional and public symbolic. Based on their findings, they pointed out that low-carbon innovation must emphasize the unique added value in the public domain, thereby evidencing the positive power of symbolic value.

#### 5.1.2. Explanations for Symbols’ Effect

The promotion effect of symbolic value on sustainable lifestyle guiding policies can be understood in terms of two aspects: self-influence and interpersonal influence. Regarding self-influence, self-construction theory posits that humans constantly construct, maintain, enhance, transform, and express themselves in social behaviors in view of the self [69]. As a common behavior, practicing sustainable lifestyle guiding policies has become a kind of resource for individuals to obtain symbolic value; that is, members of the public can construct, transform, and express themselves when practicing an environmental policy. From this perspective, the implementation of sustainable lifestyle guiding policies provides chances and paths for individuals to build, display, and distinguish themselves, which represent a key incentive factor for improving policy-experienced utility.

Regarding interpersonal influence, role identity theory posits that individuals pursue both difference and similarity when comparing themselves with others [7]. Differences can distinguish individuals from others in order to show their special group attributes and social status, while similarity can ensure that individuals achieve group expectations and avoid being excluded and punished by other group members. A sustainable lifestyle is becoming a civilized lifestyle in line with the current trend and mainstream values. The implementation of policies allows individuals to show their reputation and status, as well as convey their social role and group information to the outside world. From this perspective, implementing sustainable lifestyle guiding policies meets the needs of symbolic values such as role attributes and social status, and becomes the internal driving force and external pressure to encourage residents to engage in sustainable lifestyles.

### 5.2. Social Interaction Impact

The empirical results of the main analysis and the endogeneity test show that the association between symbolic value and *EUSLGP* was positively moderated by social interaction. Consistent with results found in this study, prior studies have found that social interaction positively impacts on pro-environmental behavior [11], and that knowledge sharing contributes to improving organizational performance [70]. Sacco and Ismail [71] found that both face-to-face interaction and computer-mediated communication can meet the needs of communication and promote positive emotions, although face-to-face interaction was more effective. It can be inferred that social interaction adjusts the target group’s attention to symbolic value, promotes the emotional experience of implementing sustainable lifestyle guiding policies, and thus improves policy-experienced utility. Studies have revealed that subjective well-being is affected by structural capital, relational capital, and cognitive capital [72], and that social interaction is positively correlated with individual involvement [73]. These findings not only support this study’s conclusions but also confirm the effectiveness of social interaction in improving policy-experienced utility.

In practice, individuals change their cognition and even existing decisions according to the information exchanged in interactions with others. Studies have revealed that social interaction leads to mutual influences between the individual and a reference group, which is an important driving force for the consistency and convergence of group behavior [74]. It can be concluded that the target group finds the symbolic value that has not been realized and masters the skills of practicing sustainable lifestyle guiding policies through exchanging information and sharing experience, which not only improves the enthusiasm of target group members to practice public policies but also changes individuals’ cognition and subjective perception of policies through the real experience of the humans around them. In addition, social interaction provides a good channel for individuals to share their emotional experiences and demonstrate their social value. If persons actively practice sustainable lifestyle guiding policies or strongly support environmental policies, they can convey environmental feelings to surrounding people through social interaction. This self-expression and social identification significantly improve policy-experienced utility, and will create a more extensive, lasting, exemplary role. Therefore, social interaction provides a new idea and path for improving the *EUSLGP* and even the general experienced utility of public policies.

### 5.3. Regional Differences

In tests of the differences between the north and south groups, the regression coefficients of symbolic value–*EUSLGP* and social interaction–*EUSLGP* were higher in the south than in the north. These findings indicate that there are significant regional differences in the effects of symbolic value and social interaction. As the southeastern provinces are more developed in terms of economy and information technology, as well as more open, compared to the northeastern provinces, residents of the former may pay more attention to symbolic values such as self-expression and relationship maintenance. In other words, the recognition and admiration of environmental behavior in the southeastern provinces provides a stronger motivation for individuals to implement sustainable lifestyle guiding policies. Moreover, the environmental governance concepts in the southeastern provinces are more advanced, and residents have higher environmental literacy and civic awareness, leading to a higher perception of policy utility.

More attention should be paid to regional differences in the influences of symbolic value and social interaction. On the one hand, more efforts should be made to promote symbolic value and social interaction in southeastern provinces. On the other hand, customized policy implementation strategies should be enacted to better activate symbolic value and social interaction in northeastern provinces based on the local economic and cultural structure. It is worth noting that the positive effects of symbolic value and social interaction on the *EUSLGP* were found in southeastern and northeastern provinces, which demonstrates the robustness of the theoretical model constructed in this study.

## 6. Conclusions, Suggestions, and Limitations

### 6.1. Conclusions

A sustainable lifestyle is crucial to achieving the UN’s sustainable development goals, and discovering the public response to sustainable lifestyle guiding policies is indispensable to change individual activities in the private sphere. In this study, a model was established by incorporating symbolic value into a model consisting of social interaction and policy-experienced utility. With Stata 16 used, ordinary least squares were applied to examine the effects of symbolic value and social interaction on the *EUSLGP.* Moreover, the robustness of the results was verified by the instrumental variable method based on two-stage least squares. There were some important findings obtained from this study. First, symbolic value positively promoted *EUSLGP* with an influence coefficient of 0.543. Second, social interaction positively moderated the association between symbolic value and the *EUSLGP*, and the effect coefficient was 0.052. Finally, significant regional differences were found in the effects of symbolic value and social interaction on the *EUSLGP*. The results found in this study indicated that symbolic value extracted from sustainable lifestyle guiding policies and interaction between social members are helpful to engage the public in practicing related policies. With the effect of symbolic value on the *EUSLGP* identified, this study fills the gap on implicit values in policy research and provides new ways to launch policies to promote sustainable lifestyles.

### 6.2. Policy Suggestions

This study found that symbolic value was positively associated with the *EUSLGP* through four aspects: self-expression, relationship consolidation, group identification, and status showing. This indicates that if policymakers endow sustainable lifestyle guiding policies with specific symbolic value through appropriate packaging, and highlight the new added value of implementing policies for satisfying the self-construction of target groups, it could help to enhance the policy-experienced utility and promote policy responses. Specifically, the symbolic values covered by sustainable lifestyle guiding policies should be deeply explored and elaborately integrated into the current trend and core national values. Labeling, demonstration, and institutionalization can be applied to enhance individuals’ identification of the symbolic values. Moreover, the government should endeavor to improve policy dissemination and publicity management. For example, new technologies such as user portraits can be used to accurately locate target groups and publish content that meets the public’s self-construction needs.

It was validated that social interaction has a positive moderating effect on the relationship between symbolic value and the *EUSLGP*, thus indicating that policy-experienced utility can be improved by targeting social interaction within the target group. A professional interactive platform should be established to encourage the public to share knowledge, experience, and emotions when practicing sustainable lifestyle guiding policies. The public should be allowed to rate the reliability of information on the interactive platform, and unreliable or even false information should be handled promptly. Furthermore, the government should set up a specific hotline to allow the public to report individuals who spread false information, to ensure trust within and between target groups and the accuracy of interactive information.

The empirical results reveal significant regional differences in the association between symbolic value and *EUSLGP*, and in the moderating effect of social interaction. Hence, the government should adapt to local conditions and introduce differentiated implementation strategies to take advantage of symbolic value and social interaction. Regional differences in the symbolic value preferences and social interactions should be grasped through surveys, thereby providing data to support the formulation of customized strategies. A segmented approach can be applied to identify the target groups and regions with the most significant associations between the variables. Priority should be given to these areas to achieve a demonstration effect that could improve *EUSLGP*. Moreover, personalized files should be created to record how symbolic value, policy-experienced utility, and social interaction are related in specific situations and conditions, so as to inform interventions to improve *EUSLGP*.

### 6.3. Limitations and Future Research

There are some possible limitations in this study. First, data were only collected in Eastern China; thus, we need to expand to samples from different regions and countries to generate more universal conclusions. Second, cross-sectional data were used to discover the relations among symbolic value, social interaction, and *EUSLGP*, which cannot establish causation or fully reflect changes in the variables over time. Consequently, the dynamic and causal relations between the variables should be addressed by using longitudinal research methods. Third, only the moderating effect of social interaction was examined. Many situational factors may influence the association between symbolic value and *EUSLGP*; these should be further studied to provide greater insight for policymakers. Finally, only the structural and relationship characteristics of social interaction were considered; more attention should be paid to establishing a systematic conceptual framework of social interaction.

## Figures and Tables

**Table 1:** Correlations, discriminant validity, and statistical value of variables.

	SV ^1^	SI ^2^	EUSLGP ^3^	DGFR ^4^
SV ^1^	**0.729**			
SI ^2^	0.034	**0.791**		
EUSLGP ^3^	0.568 **	0.357 **	**0.786**	
DGFR ^4^	0.368	0.663 **	−0.197	**0.795**
Mean	3.621	3.531	3.263	3.540
Minimum	3.288	3.125	2.842	3.158
Maximum	4.018	3.822	3.760	3.822
Standard deviation	0.736	0.653	0.557	0.416

^1^ SV: symbolic value; ^2^ SI: social interaction; ^3^ EUSLGP: experienced utility of sustainable lifestyle guiding policies; ^4^ DGFR: density of gathering with relatives and friends; bold number is the square root of AVE ($\sqrt{AVE}$); ** represents the level of significance at 5%.

**Table 2:** Regression results of symbolic value and the *EUSLGP*.

Variables	ALL	North	South
	EUSLGP ^7^	EUSLGP ^7^	EUSLGP ^7^
SV ^1^	0.543 *** (37.330)	0.495 *** (22.140)	0.575 *** (30.090)
GE ^2^	0.072 *** (3.410)	0.037 *** (2.960)	0.066 *** (3.130)
AG ^3^	0.015 (0.462)	0.023 (0.559)	0.012 (0.420)
ED ^4^	0.047 *** (4.382)	0.053 *** (4.576)	0.044 *** (4.103)
IC ^5^	−0.006 (−0.570)	−0.006 (−0.057)	−0.011 (−0.065)
FS ^6^	−0.045 *** (−0.065)	−0.039 *** (−3.413)	−0.057 *** (−4.012)
*R* ^2^	0.300	0.263	0.325
Adj-*R*^2^	0.300	0.263	0.325
F	1393.3	490.2	905.6

^1^ SV: symbolic value; ^2^ GE: gender; ^3^ AG: age; ^4^ ED: educational level; ^5^ IC: income; ^6^ FS: family size; ^7^ EUSLGP: experienced utility of sustainable lifestyle guiding policies; *** represents the level of significance at 1%; *t*-statistics in parentheses.

**Table 3:** Moderating effect test of social interaction.

Variables	ALL	North	South
	EUSLGP ^8^	EUSLGP ^8^	EUSLGP ^8^
SV ^1^	0.246 *** (8.960)	0.193 *** (4.460)	0.278 *** (7.820)
SI ^2^	0.113 * (1.960)	0.089 (0.990)	0.118 (1.580)
SV ^1^ × SI ^2^	0.052 *** (12.650)	0.051 *** (8.050)	0.053 *** (9.860)
GE ^3^	0.014 *** (3.080)	0.036 *** (3.870)	0.017 *** (3.117)
AG ^4^	0.033 (1.240)	0.024 (0.936)	0.027 (1.032)
ED ^5^	0.058 *** (3.413)	0.039 ** (2.062)	0.048 *** (2.956)
IC ^6^	−0.015 (−1.385)	−0.007 (−0.062)	−0.018 (−1.422)
FS ^7^	−0.046 *** (−3.016)	−0.052 *** (−3.220)	−0.041 *** (−2.982)
*R* ^2^	0.333	0.297	0.358
Adj-*R*^2^	0.332	0.296	0.357
F	810.8	288.9	524.6

^1^ SV: symbolic value; ^2^ SI: social interaction; ^3^ GE: gender; ^4^ AG: age; ^5^ ED: educational level; ^6^ IC: income; ^7^ FS: family size; ^8^ EUSLGP: experienced utility of sustainable lifestyle guiding policies; ***, **, and * note the level of significance at 1%, 5%, and 10%, respectively; *t*-statistics in parentheses.

**Table 4:** Endogenous test results.

Variables	Frist		Second
	SV ^1^ × SI ^8^	Variables	EUSLGP ^9^
SV ^1^	2.817 *** (29.780)	SV ^1^ × SI ^8^	0.087 *** (11.140)
DGRF ^2^	1.483 *** (7.840)	SI ^8^	0.335 ** (4.190)
SV ^1^ × DGRF ^2^	0.479 *** (35.870)	SV ^1^	0.046 (0.990)
GE ^3^	0.031 *** (2.580)	GE ^3^	0.421 *** (3.410)
AG ^4^	−0.029 (−1.434)	AG ^4^	−0.045 ** (−2.976)
ED ^5^	0.032 *** (3.072)	ED ^5^	0.042 *** (3.503)
IC ^6^	−0.016 (−1.008)	IC ^6^	−0.028 (−1.377)
FS ^7^	−0.027 *** (−2.874)	FS ^7^	−0.036 *** (−3.309)
*R* ^2^	0.808	*R* ^2^	0.318
Adj-*R*^2^	0.807	Adj-*R*^2^	0.318
F	213.8	F	124.2

^1^ SV: symbolic value; ^2^ DGRF: density of gathering with relatives and friends; ^3^ GE: gender; ^4^ AG: age; ^5^ ED: educational level; ^6^ IC: income; ^7^ FS: family size; ^8^ SI: social interaction; ^9^ EUSLGP: experienced utility of sustainable lifestyle guiding policies; *** and ** represent the level of significance at 1% and 5%, respectively; t-statistics in parentheses.

## Data Availability

Not applicable.

## Appendix A

**Questionnaire**

Dear sir or madam:

We are conducting a questionnaire survey on sustainable lifestyle guiding policies, and your response is of vital significance to us. The questionnaire is **anonymous**, **and the collected data is just for academic research. All the information in the questionnaire will not be used for any other purpose. There are no right or wrong answers**. Please read the following questions carefully and mark √ in the corresponding position according to the actual situation. Thank you for your cooperation!

1. Please select the option that best meets the following description according to your actual situation.

**No.**	**Description**	**Strongly Disagree**	**Slightly Disagree**	**Undecided**	**Slightly Agree**	**Strongly Agree**
1-1	Practicing sustainable lifestyle guiding policies show personality.	1	2	3	4	5
1-2	I can express my personality by practicing sustainable lifestyle guiding policies.	1	2	3	4	5
1-3	The image of practicing sustainable lifestyle guiding policies is line with my personality.	1	2	3	4	5
1-4	Practicing sustainable lifestyle guiding policies is consistent with my own view.	1	2	3	4	5
1-5	Practicing sustainable lifestyle guiding policies is in line with the views of my friends.	1	2	3	4	5
1-6	Practicing sustainable lifestyle guiding policies improve others’ impression of me.	1	2	3	4	5
1-7	Practicing sustainable lifestyle guiding policies is in line with the views of my relatives.	1	2	3	4	5
1-8	Practicing sustainable lifestyle guiding policies improve my relationship with others.	1	2	3	4	5
1-9	Practicing sustainable lifestyle guiding policies reflects what type of person I am.	1	2	3	4	5
1-10	People practicing sustainable lifestyle guiding policies have some similar characteristics.	1	2	3	4	5
1-11	Practicing sustainable lifestyle guiding policies makes me feel like a member of a certain group.	1	2	3	4	5
1-12	Practicing sustainable lifestyle guiding policies reflects the common interests of a certain group.	1	2	3	4	5
1-13	Practicing sustainable lifestyle guiding policies demonstrates social status.	1	2	3	4	5
1-14	Practicing sustainable lifestyle guiding policies is a symbol of social status.	1	2	3	4	5
1-15	Practicing sustainable lifestyle guiding policies enable me to gain a higher social reputation.	1	2	3	4	5
1-16	Practicing sustainable lifestyle guiding policies is a symbol of successful people.	1	2	3	4	5

2. Please select the option that best meets the following description according to your actual situation.

**No.**	**Description**	**Strongly Disagree**	**Slightly Disagree**	**Undecided**	**Slightly Agree**	**Strongly Agree**
2-1	Many people discuss sustainable lifestyle guiding policies with me.	1	2	3	4	5
2-2	Many people share with me their tips on practicing sustainable lifestyle guiding policies.	1	2	3	4	5
2-3	Close friends discuss with me sustainable lifestyle guiding policies.	1	2	3	4	5
2-4	I spend a lot of time discussing sustainable lifestyle guiding policies with others.	1	2	3	4	5
2-5	I discuss sustainable lifestyle guiding policies with others in various ways.	1	2	3	4	5
2-6	I often discuss sustainable lifestyle guiding policies with others.	1	2	3	4	5
2-7	I and people around me do not take advantage of each other’s weaknesses for own benefit when discussing sustainable lifestyle guiding policies.	1	2	3	4	5
2-8	I and people around me keep promises when discussing sustainable lifestyle guiding policies with others.	1	2	3	4	5
2-9	I and people around me answer questions honestly when discussing sustainable life-style guiding policies with others.	1	2	3	4	5
2-10	I and people around me do not pass false information to others when discussing sustainable lifestyle guiding policies.	1	2	3	4	5
2-11	I and people around me do not deceive each other when discussing sustainable lifestyle guiding policies.	1	2	3	4	5

3. Please select the option that best meets the following description according to your actual situation.

**No.**	**Description**	**Strongly Disagree**	**Slightly Disagree**	**Undecided**	**Slightly Agree**	**Strongly Agree**
3-1	The money spent on practicing sustainable lifestyle guiding policies is usually acceptable.	1	2	3	4	5
3-2	The time taken to practice sustainable lifestyle guiding policies is reasonable.	1	2	3	4	5
3-3	The energy spent on practicing sustainable lifestyle guiding policies is acceptable.	1	2	3	4	5
3-4	In general, practicing sustainable lifestyle guiding policies is worth the money.	1	2	3	4	5
3-5	Practicing sustainable lifestyle guiding policies relaxes my mood.	1	2	3	4	5
3-6	Practicing sustainable lifestyle guiding policies is an enjoyable behavior.	1	2	3	4	5
3-7	Practicing sustainable lifestyle guiding policies brings me a strong sense of spiritual pleasure.	1	2	3	4	5
3-8	Practicing sustainable lifestyle guiding policies is interesting.	1	2	3	4	5
3-9	Practicing sustainable lifestyle guiding policies helps improve the environment.	1	2	3	4	5
3-10	Practicing sustainable lifestyle guiding policies reduces environmental pollution.	1	2	3	4	5
3-11	Practicing sustainable lifestyle guiding policies brings environmental value to society.	1	2	3	4	5
3-12	Practicing sustainable lifestyle guiding policies is good for mitigating climate change.	1	2	3	4	5

4. Please select the option that best meets the following description according to your actual situation.

**No.**	**Description**	**Strongly Disagree**	**Slightly Disagree**	**Undecided**	**Slightly Agree**	**Strongly Agree**
4-1	I visit some relatives and friends regularly.	1	2	3	4	5
4-2	I often attend gatherings organized by relatives, friends, or colleagues.	1	2	3	4	5
4-3	I often communicate offline and online with relatives, friends, or colleagues.	1	2	3	4	5

5. What is your gender?

□Male      □ Female

6. What is your current age?

□ below 18  □19–25   □26–30   □31–40

□ 41–50     □51–60   □ above 60

7. What is your educational level (including those you are studying for)?

□Junior high school and below      □High school/technical secondary school

□Junior college             □Bachelor’s

□Master’s                □Doctorate

8. What is your total monthly income?

□4000RMB and below         □40001-6000 RMB

□6001-8000 RMB         □8001-10000 RMB

□10001-30000 RMB        □30000-100000RMB

□100000RMB and above

9. How many members are there in your family?

□1-2   □3   □4   □5 and above

10. Which city do you current live in?

Province _    City_

**Thank you again!**

## Appendix B

**Measurement items of variables, reliability and validity analysis.**

**Measurement Items**	**Factor Loading**				**Cronbach’s α**	**CR**	**AVE**
**Symbolic value (SV)**							
Practicing sustainable lifestyle guiding policies show personality.	**0.756**	−0.287	0.088	0.038	0.819	0.820	0.531
I can express my personality by practicing sustainable lifestyle guiding policies.	**0.738**	−0.241	0.068	0.081			
The image of practicing sustainable lifestyle guiding policies is line with my personality.	**0.742**	−0.302	−0.131	0.041			
Practicing sustainable lifestyle guiding policies is consistent with my own view.	**0.815**	−0.147	−0.138	−0.018			
Practicing sustainable lifestyle guiding policies is in line with the views of my friends.	**0.752**	−0.132	−0.106	0.031			
Practicing sustainable lifestyle guiding policies improve others’ impression of me.	**0.813**	−0.158	0.065	−0.024			
Practicing sustainable lifestyle guiding policies is in line with the views of my relatives.	**0.703**	−0.192	0.092	0.051			
Practicing sustainable lifestyle guiding policies improve my relationship with others.	**0.756**	−0.209	−0.117	0.004			
Practicing sustainable lifestyle guiding policies reflects what type of person I am.	**0.749**	−0.143	−0.237	−0.049			
People practicing sustainable lifestyle guiding policies have some similar characteristics.	**0.794**	0.109	0.165	−0.157			
Practicing sustainable lifestyle guiding policies makes me feel like a member of a certain group.	**0.830**	0.090	0.065	−0.303			
Practicing sustainable lifestyle guiding policies reflects the common interests of a certain group.	**0.799**	0.156	0.213	−0.201			
Practicing sustainable lifestyle guiding policies demonstrates social status.	**0.814**	0.075	0.204	−0.122			
Practicing sustainable lifestyle guiding policies is a symbol of social status.	**0.798**	0.007	0.205	−0.151			
Practicing sustainable lifestyle guiding policies enable me to gain a higher social reputation.	**0.755**	0.093	0.221	0.042			
Practicing sustainable lifestyle guiding policies is a symbol of successful people.	**0.743**	0.077	0.191	0.005			
**Social interaction (SI)**							
Many people discuss sustainable lifestyle guiding policies with me.	0.206	**0.867**	0.073	−0.135	0.832	0.834	0.626
Many people share with me their tips on practicing sustainable lifestyle guiding policies.	0.234	**0.765**	−0.085	−0.215			
Close friends discuss with me sustainable lifestyle guiding policies.	−0.057	**0.762**	−0.113	0.053			
I spend a lot of time discussing sustainable lifestyle guiding policies with others.	−0.049	**0.856**	−0.105	0.052			
I discuss sustainable lifestyle guiding policies with others in various ways.	−0.034	**0.792**	0.045	−0.016			
I often discuss sustainable lifestyle guiding policies with others.	−0.111	**0.789**	0.057	0.027			
I and people around me do not take advantage of each other’s weaknesses for own benefit when discussing sustainable lifestyle guiding policies.	−0.211	**0.827**	0.059	−0.063			
I and people around me keep promises when discussing sustainable lifestyle guiding policies with others.	−0.102	**0.793**	−0.041	0.013			
I and people around me answer questions honestly when discussing sustainable lifestyle guiding policies with others.	−0.031	**0.833**	−0.099	0.042			
I and people around me do not pass false information to others when discussing sustainable lifestyle guiding policies.	0.222	**0.867**	0.136	0.129			
I and people around me do not deceive each other when discussing sustainable lifestyle guiding policies.	0.308	**0.786**	0.153	0.298			
**Experienced utility of sustainable lifestyle guiding policies (EUSLGP)**							
The money spent on practicing sustainable lifestyle guiding policies is usually acceptable.	0.002	−0.077	**0.803**	0.004	0.822	0.823	0.617
The time taken to practice sustainable lifestyle guiding policies is reasonable.	−0.224	−0.179	**0.751**	0.205			
The energy spent on practicing sustainable lifestyle guiding policies is acceptable.	−0.224	−0.123	**0.789**	0.201			
In general, practicing sustainable lifestyle guiding policies is worth the money.	−0.198	−0.153	**0.864**	0.114			
Practicing sustainable lifestyle guiding policies relaxes my mood.	−0.128	−0.179	**0.719**	−0.078			
Practicing sustainable lifestyle guiding policies is an enjoyable behavior.	−0.207	−0.153	**0.832**	−0.130			
Practicing sustainable lifestyle guiding policies brings me a strong sense of spiritual pleasure.	−0.008	−0.256	**0.711**	−0.137			
Practicing sustainable lifestyle guiding policies is interesting.	0.093	−0.134	**0.789**	−0.021			
Practicing sustainable lifestyle guiding policies helps improve the environment.	0.104	0.019	**0.814**	0.177			
Practicing sustainable lifestyle guiding policies reduces environmental pollution.	0.018	0.008	**0.810**	0.143			
Practicing sustainable lifestyle guiding policies brings environmental value to society.	0.073	0.207	**0.797**	0.097			
Practicing sustainable lifestyle guiding policies is good for mitigating climate change.	−0.033	0.204	**0.748**	0.265			
**Density of gathering with relatives and friends (DGRF)**							
I visit some relatives and friends regularly.	0.042	0.217	0.02	**0.821**	0.863	0.873	0.632
I often attend gatherings organized by relatives, friends, or colleagues.	0.127	0.012	0.108	**0.815**			
I often communicate offline and online with relatives, friends, or colleagues.	0.071	0.124	0.108	**0.788**			
